# Benchmarking care outcomes for young adults with type 1 diabetes in Australia after transition to adult care

**DOI:** 10.1002/edm2.295

**Published:** 2021-09-09

**Authors:** Phidias Rueter, Kaye Farrell, Helen Phelan, Peter Colman, Maria E. Craig, Jenny Gunton, D. Jane Holmes‐Walker, the ADDN study group, ANDA study group

**Affiliations:** ^1^ Sydney Medical School University of Sydney Sydney NSW Australia; ^2^ Department of Diabetes and Endocrinology Westmead Hospital Westmead NSW Australia; ^3^ John Hunter Children’s Hospital Newcastle NSW Australia; ^4^ Department Diabetes and Endocrinology Royal Melbourne Hospital Parkville VIC Australia; ^5^ Department of Medicine University of Melbourne Parkville VIC Australia; ^6^ Institute of Endocrinology and Diabetes Children’s Hospital at Westmead Westmead NSW Australia; ^7^ Charles Perkins Centre Westmead University of Sydney Sydney NSW Australia; ^8^ School of Women’s and Children’s Health University of NSW Sydney NSW Australia; ^9^ Westmead Institute of Medical Research University of Sydney Sydney NSW Australia

**Keywords:** care outcomes, type 1 diabetes, young adults

## Abstract

**Aim:**

To determine advantages conferred by a youth‐specific transition clinic model for young adults with type 1 diabetes (T1D) at Westmead Hospital (WH) as compared with Australian registry data.

**Methods:**

Prospectively collected data included age, diabetes duration, visit frequency, post code, BMI, mode of insulin delivery, continuous glucose monitoring, HbA1c, albumin creatinine ratio, BP, retinopathy and diabetic ketoacidosis (DKA) for all WH T1D clinic attendees aged 16–25 between January 2017 and June 2018 (*n* = 269). Results were compared with data collected during the same time period from 2 separate Australian data registries, one longitudinal (Australasian Diabetes Data Network, ADDN) and one a spot survey (the Australian National Diabetes Audit, ANDA).

**Results:**

Across the three cohorts, HbA1c was similar (respectively, WH, ADDN, ANDA; 8.7%[72mmol/mol], 8.7%[72mmol/mol], 8.5%[69mmol/mol]) and HbA1c was significantly higher in young adults <21 years (8.7–8.9%[73‐75mmol/mol]) as compared with ≥21 years (8.5%[69mmol/mol], *p *< .002). In the WH cohort, median interval between visits was shorter than in ADDN (4.5 vs. 9.0 months) and DKA was lower (respectively, 3.6 and 9.2/100 patient years; *p *< .001).

**Conclusions:**

While suboptimal HbA1c was recorded in all centres, the WH model of care saw increased attendance and reduced admissions with DKA as compared with other Australian adult centres.

## Abstract 2

A model of care for young people with type I diabetes as they transition to adult care was benchmarked with two separate Australian data registries. Similar glycaemic outcomes were achieved nationally but the care model achieved a marked reduction in diabetic ketoacidosis and increased clinic attendance frequency.

## 1 INTRODUCTION

In June 2017, at the time of the study, 1.25 million Australians were known to have diabetes, with 118,845 of those having type 1 diabetes (T1D).^1^ Of these, 27,236 (23%) were <30 years old.^2^ Transition clinics are increasingly utilized to provide assistance for the young person with diabetes to navigate the move from paediatric to adult care. This period of life characterized by significant physiological and psychological changes of adolescence^3^ is also associated with higher average HbA1c.^4^, ^5^ Transition in Australia typically occurs on completion of schooling (age 16–18) or at age 18. In NSW, newly diagnosed T1D patients aged ≥15 years are managed in adult hospitals while in other states when age is ≥16. Improvements in the transition process can be translated to prevention of loss to follow‐up and improved diabetes care outcomes.^6^ Yet transition from more family‐oriented paediatric practice to adult practice, where independent and self‐directed diabetes management is required, can result in loss of engagement with the adult healthcare team if the young person is not prepared for the differences in care.^7^ The resulting reduced frequency of clinic visits and deterioration in glycaemia correlate with an increased incidence of acute and, prevalence of, chronic diabetes‐related complications.^8^, ^9^ Ensuring attendance at scheduled visits, building connections between paediatric and adult care providers and prevention of loss to follow‐up are the most important factors for successful transition.^10^, ^11^

Treatment adherence and optimized glycaemia align with improved patient quality of life and decreased risk of diabetes‐related complications, as reflected in long‐term trials.^12^ Reducing hospital admissions is key to improving quality of life of young people with diabetes with additional cost benefits, given in‐hospital care accounts for up to 40% of total cost of treating diabetes.^13^ To date, no Australia‐wide benchmarking study comparing the impact of transition to adult care on diabetes outcomes in the 16–25 year age group has been conducted.

The young adult diabetes clinic at Westmead Hospital (WH), Sydney, Australia, established in 2001,^6^ cares for more than 300 young people with T1D. The model of care employed by the clinic at WH was unique in Australia when initiated in 2002 and is based around a dedicated full‐time transition coordinator/diabetes educator and a youth‐friendly clinic environment with clinic hours tailored to young participants.^6^ Attendees are rebooked for appointments every 3 months, with complications screening for retinopathy, neuropathy and nephropathy performed annually, as part of the routine clinic visit. Few clinics in Australia have a dedicated diabetes transition coordinator who can actively follow‐up those who have missed appointments. Young adults attending the WH clinic are actively reminded of their appointments through an SMS text reminder service and are offered rescheduling of missed appointments within 4 weeks to improve attendance at follow‐up^6^ and have access to an after‐hours telephone support service provided by the transition coordinator for sick day management according to previously published protocols.^14^, ^15^ While many diabetes services across Australia now separate young people with diabetes into a ‘transition clinic’, distinct from clinics for adults with diabetes, the clinics do not offer extended hours, do not have active surveillance of attendance and do not offer direct access to after‐hours phone support.

Screening rates for diabetes‐related complications offer a means of benchmarking service effectiveness in diabetes care. Diabetes‐related complications can be divided into:

1. Acute complications such as hyperglycaemia [with or without diabetic ketoacidosis (DKA)] and severe hypoglycaemia resulting in hospital admissions or ambulance call outs. Acute complications such as DKA occur with increased frequency in young people with diabetes.^14^
2. Chronic complications include albuminuria, retinopathy, macro‐ and microvascular disease, neuropathy and hypertension. These are often first detected in young people after transition to adult care but screening in this age group is generally suboptimal^8^, ^9^

Benchmarking studies and comparisons between different models of care help to identify factors to improve the quality of care provided to participants, thereby facilitating improved clinical outcomes.^16^ The aim of this benchmarking study was to determine whether the model of care provided for young people with T1D attending WH improved the frequency of attendance, complications screening and acute care outcomes, as compared with other centres providing care for young adults with T1D across Australia across a diversity of clinical settings. Two separate Australian diabetes data registries were used for benchmarking care outcomes.

## 2 MATERIALS AND METHODS

### 2.1 Ethics

Ethics approval was obtained by the Western Sydney Local Health District Human Research Ethics Committee as a quality assurance activity (SAC2018/3/6.9 (5585) QA).

### 2.2 Subjects

This retrospective cohort study included all attendees aged 16–25 with T1D of the transition clinic at WH with all visits recorded between 1st Jan 2017 and 30th June 2018 (WH cohort), all registrants aged 16–25 attending adult hospitals with at least one visit recorded in the Australasian Diabetes Data Network (ADDN) between 1^st^ Jan 2017–30th June 2018 (ADDN cohort), and all participants with T1D aged 16–25 attending adult centres over 4 consecutive weeks in June 2017 who contributed single visit data to the Australian National Diabetes Audit (ANDA) “spot survey” (ANDA cohort). Data were excluded for participants who were pregnant during the study period or within 6 months of diabetes diagnosis. Data from WH participants were excluded from ADDN and ANDA data extractions.

### 2.3 Data collection

Individual data from WH, ANDA and ADDN were deidentified with a separate record being kept for reidentification stored locally at WH and ADDN participating centres. Data were password protected and stored on department servers, with password and provider access granted exclusively to individuals involved in the research at participating centres. ANDA data were not able to be reidentified.

Data collected at WH included age, duration diabetes, post code of residence, visit frequency, Insulin regimen (continuous subcutaneous insulin infusion, CSII, or multiple daily injections, MDI), CGM usage for >3 months in the time period of interest (as the majority who discontinued using CGM ceased within the first month of use), HbA1c at each visit, blood pressure, spot urine albumin/creatinine ratio and retinal assessment. Retinal assessments at WH were conducted at the clinic the patient attended by direct ophthalmoscopy or recorded from formal ophthalmology assessment performed by optometrist or ophthalmologist. Lipid data and data about foot examination were not taken into account, as they were largely unrecorded in clinic notes. BMI data were not consistently recorded at WMH due to the concerns about body image, common in those transitioning from paediatric care.^17^

Deidentified data obtained from ADDN included ADDN identifier, date of birth, gender, country of birth, post code, diabetes type, date of diagnosis, date of visit, HbA1c, height, weight, systolic BP, diastolic BP, insulin regimen, CGM used (yes/no), DKA episodes and albumin creatinine ratio. There was no available retinopathy data.

Deidentified data obtained from ANDA included age at visit, gender, date of diagnosis diabetes, type of diabetes, insulin regimen, blood pressure, attended optometrist/ophthalmologist (yes/no), retinopathy (present/absent), laser treatment (yes/no), urinary albumin/creatinine ratio or excretion rate result, HbA1c result and number of visits in last 12 months. There was no DKA data collected by ANDA. Retinopathy screening rates were not specifically recorded, rather positive results were recorded.

For all sites, DKA presentations were validated by treating physician from hospital records for WH and for ADDN were validated by the registry coordinator at each participating hospital. DKA is only entered in ADDN If it is ≥1 and the validation process identified both missing DKA events and incorrectly recorded DKA events. All participating centres validated DKA data.

ADDN is a longitudinal database into which 6 transition clinics based in tertiary adult referral hospitals (Appendix) from 4 different states across Australia (excluding NSW where the WH clinic is located) uploaded information in the time period of interest. Details of methodology used for data collection and storage have been previously published.^5^

The ANDA survey is conducted biannually by the National Association of Diabetes Centres (NADC) under the auspices of the Australian Diabetes Society (ADS) and is funded by the Australian Government.^18^ It collects data from a variety of care delivery settings including primary, secondary and tertiary centres across all states and territories. The survey collects data on diabetes treatment and complications and was conducted in June 2017 coinciding with the period of audit. ANDA collects clinical information at a single time point but includes information up to 6 months prior to the audit date. The participating centres (49 of 62 participating centres) were all adult healthcare settings (Appendix). Centres which did not collect clinical outcomes for the age group of interest (13 centres) were excluded. Individual centres were not able to be identified or reidentified in ADDN.

The age and duration of diabetes of participants were calculated at 1^st^ January 2017. The visit frequency was calculated as 18 months divided by the number of visits. Visit frequency was only available for comparison between ADDN and WH, as the ANDA survey did not collect longitudinal data. BMI was calculated from patient weight (kg)/(height, (m))^2^.

Where there was more than one HbA1c value over the 18‐month time period the median value for an individual was used for WH and ADDN cohorts. At WH, HbA1c was measured using point of care testing (Alere Afinion AS100 Analyzer, Axis‐Shield PoC AS, Norway) at each visit if >2.5 months since the previous test. Insulin administration was recorded as either continuous subcutaneous insulin infusion (CSII) or multiple daily injections (MDI). Government funded continuous glucose monitoring (CGM) for individuals <21 years was introduced in Australia in March 2017. CGM usage was recorded if used for 3 months or more during the study and was recorded for the WH and ADDN cohorts. Usage was recorded as Yes/No but percentage usage data was not recorded reliably by participating centres.

Median incomes were determined from the 2017–2018 Australian Bureau of Statistics income data^19^ using individual postcodes as recorded in the WH and ADDN cohorts (not available for the ANDA cohort).

### 2.4 Statistical analysis

For analyses, demographic, outcome and complications data were separated into three groups WH, ADDN and ANDA. The distributions of continuous data were checked for skew by graphing and using one‐sample Kolmogorov‐Smirnov testing. Where skew was detected, data across the three groups were compared using the non‐parametric Kruskal‐Wallis test, and if group differences were found, post hoc pairwise comparisons using Mann‐Whitney U tests were undertaken between WH and the relevant group. Where a statistically significant difference was observed across the three groups, post hoc pairwise comparisons were performed between groups of interest. For normally distributed data, means were compared by ANOVA, and if group differences were observed, post hoc pairwise comparisons were performed with independent samples *t* tests. Categorical data (eg use or non‐use of CSII) were compared across using chi‐square testing. Spearman correlation analysis was performed between skewed continuous and/or ordinal variables (eg number of DKA admissions and time between visits). Significance was set at *p*‐values <.05, and all analyses conducted using IBM SPSS Version 25 (IBM Corporation. IBM SPSS Statistics for Windows. 25.0 ed. IBM Corporation). All analyses were based on raw data with no adjustment for risks.

## 3 RESULTS

A total of 314 participants from WH, 472 from ANDA and 988 from ADDN were included in the study. ADDN data were extracted from the ADDN data ENQ0042 and were received on the 24^th^ December 2018. The ANDA data extraction was performed 29th October 2018. Following exclusions, there were 269 participants from WH, 385 from ANDA and 950 from ADDN. Six centres contributed data to ADDN, while 49 of the 64 ANDA centres contributed data in the age group of interest (Table 1). Gender distribution was similar across the three groups. There was no difference in age between the groups. The mean duration of diabetes was significantly longer in the WH cohort compared with other centres. BMI data were not recorded for the WH cohort, BMI was not significantly different between the ADDN and ANDA cohorts. Visit frequency (Table 2) was significantly higher at WH, with a median interval between appointments of 4.5 months (3.6–6.3 months), compared to a median interval between appointments of 9.0 months (3.6–18 months) in ADDN centres.

**TABLE 1:** Demographic characteristics

Centres	WH	ADDN	ANDA
Number of centres	1	6	49
Number of participants	269	950	385
Proportion male: female (%)	46:54	51:49	51:49
Age (years)	20.3	20.2	20.3
Proportion Age <21 (%)	52	57	54
Duration diabetes (years)	11.6	10.7*	9.7**
BMI (kg/m^2^)	N/A	25.1	24.1
BMI not recorded (%)	100	11	3

*p < .05, **p< 0.001.

Abbreviation: N/A not available.

**TABLE 2:** Outcomes

Centres	WH		ADDN centres		ANDA centres	
Participants number (n)	269		950		385	
Interval to visit (months)	4.5		9.0**		N/A	
HbA1c (%/ mmol/mol)	8.7	72	8.7	72	8.5	69
HbA1c missing n (%)	1 (0)		283 (30)		30 (8)	
CSII (%)	47		29 **		31 **	
HbA1c <7.5% (58 mmol/mol) (%)	16		13		20	
HbA1c male (mmol/mol)	8.6	70	8.8	73	8.8	70
HbA1c female (mmol/mol)	8.8	73	8.5	69	8.5	69
HbA1c MDI (%/ mmol/mol)	8.8	73	8.9	74	8.6	70
HbA1c CSII (%/ mmol/mol)	8.6	70	8.4#	68#	8.3	67
CGM HbA1c (%/mmol/mol)	8.8	73	8.8	73	N/A	N/A
No CGM HbA1c	8.7	72	8.7	72	N/A	N/A
HbA1c 16‐<21 years (%/ mmol/mol)	9.0	73	8.9	74	8.8	73
HbA1c 21–25 years (%/ mmol/mol)	8.5#	69#	8.5#	68#	8.5	69#

All HbA1c values are medians. *p<0.05, **p< 0.001, # *p*<0.05 within data set analysis.

Abbreviation: N/A, not available.

Median HbA1c was similar across the three cohorts at 8.5–8.7% (69–72 mmol/mol; Table 2) but was significantly higher in those aged 16‐<21 than in those aged 21–25 years (*p *= .002) in each of the three cohorts (within group analysis). There were no differences in HbA1c by gender. There was no difference in the proportion meeting the previous recommended targets for young adults, HbA1c <7.5% (58mmol/mol), across the three data sets (range 13%–20%).

The proportion of participants used CSII in the ADDN centres was lower compared to WH cohort (29 vs. 47%; *p *= .002). Those using CSII had consistently lower HbA1c than MDI users in each cohort (WH/ADDN/ANDA respectively,‐0.2% [−2.1 mmol/mol] −0.5% [‐ 5.5mmol/mol]; −0.3% [−3.3mmol/mol; *p *< .001). Median interval between appointments for MDI users and CSII users for the WH cohort was 4.5 months and 4.25 months, respectively, (*p *< .05) and in the ADDN cohort 8.0 months and 9.0 months, respectively (NS).

In participants using CGM for >3 months, HbA1c did not differ significantly from those not using CGM but details on percentage use of CGM were not available. There was no difference in DKA frequency with CGM use.

Admissions with DKA (Table 3) were significantly lower in the WH cohort than in the ADDN cohort (3.6/100 vs 9.2/100 person years, *p *< .001). DKA admission data were not collected in the ANDA survey. An increased likelihood of admission with DKA was associated with longer interval between visits (*p* < .001) and with HbA1c >9% (75mmol/mol) (*p* < .05), but there was no association with mode of insulin delivery (CSII cf MDI; *p *= .48) from analysis of the ADDN cohort. There were insufficient DKA admissions in the WH cohort to assess correlations with DKA admission.

**TABLE 3:** Complications of diabetes

Centres	WH	ADDN centres	ANDA centres
Participants number (n)	269	950	385
Proportion ACR screened (%)	59	43**	51*
Abnormal ACR, >3mg/mmol (%)	4.5	3.6	0.8**
Blood Pressure measured (%)	92	61*	80*
Systolic BP mmHg (mean)	122 ± 11	117 ± 12*	118 ± 13*
Retinopathy screened (%)	80	N/A	54**
Retinopathy present (%)	5.9	4.3	5.5
Admissions with DKA (n individuals)	14 (9)	132 (70)*	N/A
DKA per 100 person years	3.6	9.2*	N/A

Abbreviations: ACR, albumin/creatinine ratio; N/A, not available.

*p<0.05, **p< 0.001.

There was a significantly greater proportion with blood pressure recorded (Table 3) in the WH cohort than ADDN and ANDA (respectively, 92/61/80%, *p *< .001). The WH cohort showed higher mean systolic blood pressure compared with the other cohorts (*p *< .001). The proportion of individuals screened for albuminuria was higher at WH than the other centres (59/43/51%, *p *< .05) but screening rates were low in all cohorts. Both WH and ADDN cohorts had similar proportions of individuals with elevated urine ACR (defined as >3.5 mg/mmol) (4.5% vs. 3.6%), and however, ANDA had a significantly lower proportion with 0.8% having elevated levels (*p *< .001). Presence of diabetic retinopathy was similar in both the WH and ANDA databases (5.5% vs. 5.9%, *p *= .86) but proportion screened for retinopathy in the previous 12 months was greater in the WH cohort (80 vs 54%; *p *< .001). While retinopathy presence was recorded in the ADDN cohort, proportion screened was not able to be determined due to incomplete data.

Median incomes were similar across the WH and ADDN groups ($AUD 48 018 vs. $AUD 46 923). A small difference was found between those using CSII and MDI in the WH cohort only ($AUD 49 543 vs. $AUD 47 825 *p *< .05) but not in the ADDN cohort. Median income did not differ by HbA1c.

## 4 DISCUSSION

This is the first report of glycaemic outcomes in young adults with T1D following transition in Australia. In this study, we have used existing Australian data registries to evaluate service performance of a youth orientated diabetes transition service at WH for people with T1D aged 16–25 with several unique features not replicated in other adult centres in Australia. While the two registries used for benchmarking were different, a number of findings were similar across the three cohorts. Moreover, the findings were also similar to reports from international registries.

In Australia, across the three study populations, HbA1c values were consistent. Median HbA1c of 8.7% (72 mmol/mol) in WH and ADDN cohorts and 8.5% (69 mmol/mol) for ANDA were comparable with international registries, reflecting the difficulty of achieving optimal HbA1c in the 16‐ to 25‐year‐old age group for many psychological, social and developmental reasons.^3^, ^20^ Values obtained in Australia lie between the values reported for the T1D Exchange Registry in the United States, 9.2% (77 mmol/mol) and the DPV registry in Germany/Austria, 8.2% (65 mmol/mol).^21^ The HbA1c in the ANDA cohort represented a single measure, whereas the HbA1c for ADDN and Westmead cohorts reflected the median value for all visits of an individual in an 18‐month period. The ANDA registry covers a more diverse group of treatment centres (Appendix) whereas ADDN centres and WH were all tertiary referral hospitals.

The significantly lower HbA1c found in the WH and ADDN participants aged 21–25 years has similarly been observed by the DPV and T1D Exchange Registries although the age grouping of 12–18 and 18–30 was slightly different^21^ in the latter registries. Overseas registries have found lower HbA1c in CSII users^4^, ^20^ which was similarly observed in CSII users from the ADDN but not the WH cohorts despite a greater proportion of CSII users in the WH cohort than in the ADDN cohort. There was no impact of socioeconomic status on glycaemic outcomes, as assessed by median household income from the WH and ADDN cohorts. The addition of CGM did not appear to impact glycaemic outcomes; however, CGM was progressively introduced over 15 months of the study period and data was not available on percentage use with previous studies showing improvement in glycaemic outcomes only with use >80%.^22^ In Australia, funding for CGM for patients under the age of 21 became available on the 1st April 2017, and an internal audit at WMH indicated that by September the pick‐up rate was lower than 10%.

Across the three Australian cohorts, between 13 and 20% attained a target HbA1c of <7.5% (58 mmol/mol) similar to that in the US T1D Exchange (21%).^4^ In other publications, higher HbA1c is associated with increased risk for admission with DKA.^23^, ^24^ Despite the similarities in HbA1c between the two longitudinal cohorts, ADDN and WH, two key differences from the Westmead model of care were apparent: reduced DKA admissions and shorter interval between appointments.

Presentations with DKA over the 18 months in the WH cohort were significantly lower than in the ADDN cohort. DKA is the most common avoidable cause of hospitalization in the 15‐ to 25‐year‐old age group. Presentation with DKA in the ADDN cohort was associated with higher HbA1c and longer interval between visits. Access to after‐hours phone support in the WH cohort has previously been shown to reduce DKA admissions as compared with young adults with T1D who do not have access to this support.^15^ The after‐hours phone support has previously been shown to be used by those who attended clinic more frequently, possibly due to increased awareness of the service. Previous analysis at WH found a cost saving of $250,000 per annum from reduced DKA admissions and reduced length of stay for those who attended the young adult diabetes service at Westmead with access to phone support.^15^, ^24^ The National Weighted Activity unit cost of a DKA admission in Australia is $AU4400. The difference therefore between WH and ADDN services based on number of DKA admissions per 100 patient years is $25,000 per 100 patient years offsetting costs associated with after‐hours phone support.

Complications screening records were more complete in the WH cohort. Screening from puberty onwards is critical, as puberty has a substantial impact on the development of diabetes complications^25^ and early detection of complications to slow or prevent progression of complications is highly cost‐effective.^26^ The WH cohort had higher systolic blood pressure and higher rates of albuminuria but also a longer median duration of diabetes. Higher systolic blood pressure in the WH cohort may be related to comorbid high rates of obesity seen in Western Sydney,^27^ and however, as weight was not routinely collected in the WH cohort, this could not be confirmed. The lower ACR in the ANDA population, conversely may be related to lower systolic BP and shorter duration of diabetes. The complications data, however, should be treated with caution in view of incompleteness of data collection in all three cohorts and low rates of diabetes‐related complications. Risk adjustment was not performed as data was not sufficiently complete. The results highlight the critical need for improved surveillance and reporting of diabetes‐related complications in young adults with diabetes in Australia across all states. Prevalence rates for retinopathy and albuminuria of 5%, despite low screening rates, indicate the need for commencement of preventative therapies in young adults.

While a few hospitals offer dedicated transition coordinators in Australia,^28^ their sample size was small. None of the centres contributing to the ADDN data set had a dedicated transition coordinator. The number of ANDA centres with transition coordinators is unknown but individual centre numbers again were small and unlikely to have impacted the group analysis.

Transition coordinators are increasingly a point of focus for enhancing transition outcomes.^24^, ^28^ A deeper analysis of cost‐benefit of transition coordinators in conjunction with an assessment of the perceived patient experience would be valuable.

This is the first analysis of care outcomes for young people with T1D managed in adult centres across Australia. Data have been published comparing care of young people with T1D in metropolitan and regional New South Wales^28^ and for T1D aged <18 managed in paediatric centres.^29^ Here we have shown in Australia‐wide data sets, with good representation, that there is evidence of consistency of, albeit suboptimal, care outcomes across different settings. The WH cohort represents approximately 60% of all young adults with T1D in the referral area of WH but is unknown for other centres in ADDN and ANDA. The ADDN registry (adult centres) provided comparable service delivery in tertiary facilities in metropolitan centres. There are no comparable measures for young adults who do not attend adult diabetes‐specific healthcare providers but a previous publication suggests they may be overrepresented in DKA admissions, compared with those who regularly attend transition clinics.^24^

Comparison of models of care used in transition clinics for young adults with T1D would aid in determining specific factors contributing to improved care outcomes, particularly reduction in DKA admissions and consistency and frequency of follow‐up after transition to adult care, two hallmarks of the young adult diabetes service at Westmead. Weaknesses identified in all of the data sets include a lack of reporting of impact of diabetes on mental health, staffing levels for each of the services and staff training in care or youth at transition. This would be a valuable area for future study. The ADDN registry has now expanded to include additional adult centres, with potential to gain greater understanding of which centres are achieving improvements in care outcomes over time. More detailed analysis of models of care across Australia is required to identify specific factors which improve care outcomes.

While there was some overlap of data for the ANDA and ADDN registries, ANDA was a spot survey and the overlapping data represented only a small proportion of all ANDA data collected, with only 3 of 49 ANDA centres contributing to both registries. Due to the de‐identification process used for ANDA data, it was not possible to determine the extent of overlap. The ANDA database was not longitudinal and could therefore not be compared adequately to the other databases, and furthermore, it did not include DKA admissions. There were significant missing data in both the ANDA and ADDN databases for complications screening. No risk adjustment has been performed for any of the outcomes reported due to confounding from incomplete data collection.

Improved screening and increased identification of diabetes‐related complications would allow more in‐depth analysis of factors contributing to care outcomes. The potential to combine data from ADDN and ANDA registries in the future would strengthen the ability to perform analysis of adjusted risk and identify centres providing models of care which improve glycaemic outcomes and acute and chronic complications for young people with T1D.

The main finding of the study was increased attendance and a significant reduction in DKA admissions, despite suboptimal glycaemia, through provision of a dedicated diabetes care coordinator/diabetes educator, low cost interventions such as appointment reminders and rebooking of missed appointments, extended clinic hours to improve attendance and direct access to out of hours phone support for sick day management. Further research is required to compare pathways of care across the different care providers contributing data to each registry and identify other components of care which could be implemented at low cost across all care providers. Improvements in glycaemia, prevention of acute complications and screening for diabetes‐related complications is paramount in young people with T1D to prevent progression of complications.

## CONFLICT OF INTEREST

All authors declare there are no conflicts of interest real or perceived in relation to this work.

## AUTHOR CONTRIBUTIONS

Phidias Rueter: Data curation, Formal analysis, Writing original draft. Kaye Farrell: Data curation, Writing review and Editing. Helen Phelan: Conceptualization, Writing review and editing. Peter Colman: Conceptualization, Writing review and Editing. ADDN study group representative. Maria E Craig Conceptualization, Writing review and Editing ADDN study group representative. Jenny Gunton: Conceptualization, Formal analysis, Validation, Writing review and Editing, ANDA study group representative. D Jane Holmes‐Walker: Conceptualization, Methodology, Supervision, Validation formal analysis, Writing original draft and review on behalf of the ADDN study group: Holders of data repository and approve clinical studies using data from repository. ANDA study group: Holders of data repository and approve clinical studies using data from repository.

## DATA AVAILABILITY STATEMENT

Associate Professor Jane Holmes‐Walker and Dr Phidias Rueter are the guarantors of this work and, as such, have full access to all the data in the study and take responsibility for the integrity of the data and the accuracy of the data analysis.
