# Supplementary material for: Benchmarking care outcomes for young adults with type 1 diabetes in Australia after transition to adult care
Source: Endocrinol Diabetes Metab. 2021 Sep 9;4(4):e00295. doi: 10.1002/edm2.295 (PMC8502218; doi:10.1002/edm2.295)
Supplement: Supplementary file 1 [file EDM2-4-e00295-s001.docx]

Appendix
List of ANDA Centres

| ACT Health Diabetes Service  Albury Wodonga Aboriginal Health Service  Albury Wodonga Health  Austin Health  Baker Heart and Diabetes Institute  Ballarat Health Services, Diabetes Education     Clinic  Bankstown-Lidcombe Hospital, Diabetes     Centre  Barwon Health, Diabetes Referral Centre  Beechworth Health Service  Benalla Community Health  Bendigo Diabetes and Endocrine Centre  Bendigo Health   Blacktown Hospital Diabetes Centre  CAHS Perth, Diabetes Service  Cairns Diabetes Centre  Castlemaine District Community Health Centre  Cobram District Health  Eastern Health  Frankston Hospital, Diabetes Centre  Gardens Medical Group  Gateway Health, Wangaratta  Gateway Health, Wodonga  GNS Diabetes Service  Gold Coast University Hospital  GV Health Diabetes Centre  GP Plus Noarlunga, Intermediate Care Diabetes     Services  Healthy Living NT, Alice Springs  Healthy Living NT, Darwin  Ipswich Diabetes Service  John Morris Diabetes Centre, NICS, Launceston     General Hospital  Kyabram District Health Service  Liverpool Diabetes and Endocrine Service  Logan Beaudesert Diabetes Service  **Lyell McEwin Hospital** | Macarthur Diabetes Service  Monash Health, Clayton  Monash Health, Dandenong  Mount Druitt Hospital, Diabetes Centre  Mrumbidgee Local Health District  Nathalia District Hospital/Primary Care     Connect Northern Health  Numurkah District Health Service  Princess Alexandra Hospital  Prince of Wales Hospital, Diabetes Centre  Queensland Diabetes and Endocrine Centre,    Mater Health Redland Hospital & Health Service  Rockingham General Hospital  Royal Hobart Hospital  **Royal Melbourne Hospital**  Royal North Shore, Diabetes Education,     Dept. of Endocrinology  Royal Perth Hospital  Royal Prince Alfred Hospital, Diabetes Centre Seymour Health  South West Hospital & Health Service  **St Vincent’s Public Hospital, Melbourne**  St Vincent’s Hospital, Darlinghurst, Diabetes     Centre  Sunshine Coast Diabetes and Endocrinology     Service  Tallangatta Health Service  The Alfred, Dept. of Endocrinology and   Diabetes  Townsville Hospital, Diabetes and Endocrine     Centre  Tweed Diabetes Centre  Western Health  ~~Westmead Hospital, Diabetes and     Endocrinology~~ |
| --- | --- |

*N.B. Data from Westmead was* ***excluded. Highlighted hospital contributed data to both ADDN and ANDA but represented a small percentage of the whole***

List of Adult ADDN Centres at time of report

Royal Melbourne Hospital, Melbourne, Victoria

Lyell McEwin Hospital, Adelaide, South Australia

Fiona Stanley Hospital, Perth, Western Australia

Mater Hospital, Brisbane, Queensland

St Vincent’s Hospital, Melbourne, Victoria

Sunshine Hospital, Melbourne, Victoria
